# Determinants of Infodemics During Disease Outbreaks: A Systematic Review

**DOI:** 10.3389/fpubh.2021.603603

**Published:** 2021-03-29

**Authors:** Javier Alvarez-Galvez, Victor Suarez-Lledo, Antonio Rojas-Garcia

**Affiliations:** ^1^Department of Biomedicine, Biotechnology, and Public Health, University of Cadiz, Cadiz, Spain; ^2^School of Public Health, Imperial College London, London, United Kingdom; ^3^Department of Applied Health Research, University College London, London, United Kingdom

**Keywords:** infodemics, social media, misinformation, epidemics, outbreaks

## Abstract

**Background:** The widespread use of social media represents an unprecedented opportunity for health promotion. We have more information and evidence-based health related knowledge, for instance about healthy habits or possible risk behaviors. However, these tools also carry some disadvantages since they also open the door to new social and health risks, in particular during health emergencies. This systematic review aims to study the determinants of infodemics during disease outbreaks, drawing on both quantitative and qualitative methods.

**Methods:** We searched research articles in PubMed, Scopus, Medline, Embase, CINAHL, Sociological abstracts, Cochrane Library, and Web of Science. Additional research works were included by searching bibliographies of electronically retrieved review articles.

**Results:** Finally, 42 studies were included in the review. Five determinants of infodemics were identified: (1) information sources; (2) online communities' structure and consensus; (3) communication channels (i.e., mass media, social media, forums, and websites); (4) messages content (i.e., quality of information, sensationalism, etc.,); and (5) context (e.g., social consensus, health emergencies, public opinion, etc.). Studied selected in this systematic review identified different measures to combat misinformation during outbreaks.

**Conclusion:** The clarity of the health promotion messages has been proven essential to prevent the spread of a particular disease and to avoid potential risks, but it is also fundamental to understand the network structure of social media platforms and the emergency context where misinformation might dynamically evolve. Therefore, in order to prevent future infodemics, special attention will need to be paid both to increase the visibility of evidence-based knowledge generated by health organizations and academia, and to detect the possible sources of mis/disinformation.

## Introduction

The COVID-19 pandemic has been followed by a massive “infodemic”, which has been recently defined as “an over-abundance of information – some accurate and some not – that makes it hard for people to find trustworthy sources and reliable guidance when they need it” (1). As a consequence, the excessive amount of information concerning the virus SARS-CoV-2 and the COVID-19 disease is making more difficult the identification and assessment of possible solutions. In this context, the frontiers between evidence-based knowledge, anecdotal evidence and health misinformation has become more diffuse (2, 3). The massive diffusion of health information in traditional and new media represents a serious problem due to the excess of noise (i.e., understood as an overabundance of signals about a certain topic), but also to the incorrect criteria of opinion leaders that can contribute to the development of misconceptions and risk behaviors which might subsequently alter the effectiveness of government and health authorities countermeasures (3).

Although, the formation of collective opinions is a widely studied theme in social sciences like sociology, political sciences or communication (4), this topic has recently gained special relevance in other areas of study such as health sciences (5). The new interest in this research topic is associated with the use and extension of Information and Communication Technologies (ICT) for health promotion and, in particular, with the progressive proliferation of health misinformation in social media platforms such as Twitter, Facebook, Instagram, WhatsApp, or YouTube. Today, these new tools have been incorporated in multiple spheres of our daily life, so they have radically changed our lives and the forms we interact with our peers (6). The way we currently communicate, share, receive, use, and search for both general and health-specific information has been deeply altered in the last 20 years. At present, 40% of the world population has access to Internet, the global expansion of social media covers around 39% of world population and 1.5 billion people use mobile devices to have instantaneously access to internet (7). In the EU context, 75% of European citizens considered the ICT as a good tool for finding health information (8). However, recent studies have showed that 40–50% of websites related to common diseases contained misinformation (9–11), and social media are also contributing to spread fake news on different health topics (5).

Research evidence show that the widespread use of social media represents an unprecedented opportunity for health promotion (12). We have more access to information and health-related knowledge than ever before regarding healthy habits, social and economic determinants of health, possible risk behaviors and health promotion (5). Currently, the accessibility to health contents has dramatically increased and we can find health information across multiple sources: health forums, thematic channels, direct (online) access to health experts or agencies, news online, or social media (13). Simultaneously, there is a greater availability of public data and contents about opinions, attitudes and behaviors that are continuously generated online and can be useful for encouraging healthy habits. In addition, the online expansion of health related knowledge make possible for patients to access and share medical information with other peers, acquire self-efficacy in fulfilling treatments and increase adherence in therapies and treatments (12, 14–18). However, these tools also bring some disadvantages since they also open the door to new social and health risks (19, 20). For instance, the lack of control of health information on the Internet indicates the current need to regulate the quality and public availability of health information online (21). Furthermore, the unequal access to information and the development of abilities for using new media can produce inequalities in the accessibility to health-related information, and therefore in health and social well-being of population (6, 8). The spread of self-medication cases, the proliferation of miracle diets and treatments, the anti-vaccine movements and the growing vaccine hesitancy, uninformed decision making about health-related questions, and inexpert diagnosis are some of the common risks associated to the use of new media (22).

Despite advantages may apparently be greater than disadvantages, some disease outbreaks such as the H1N1, Ebola, or Zika showed that, in times of social emergency, misinformation can provoke serious consequences, since the mechanisms of social influence can amplify fears during epidemics (15, 16). Although the concept of infodemics has only recently been defined, fear and massive misinformation through social platforms have undermined the actions taken to tackle outbreaks during the last two decades (23, 24). As an example, regarding the H1N1 or the Ebola epidemics, the misinformation phenomenon on social media has fostered unfounded myths and fears about these epidemics (25). The recent outbreak of Zika virus infections in South and Central America has also led to significant sustained myths and rumors about its pathophysiology, prevention and possible treatments that have captured massive public attention (26, 27). This problem has become more evident during the COVID-19 pandemic where the unknown social and health emergency context has brought an excess of information never before known, which is ultimately hindering the search and finding of solutions for the adequate control of the pandemic. Therefore, the determinants of infodemics during outbreaks are becoming a major priority for national governments and international health organizations in these days (3).

In order to fill this knowledge gap, the present work aims to study the determinants of infodemics during disease outbreaks. Specifically, this literature review seeks to (1) identify the factors that make possible the spread of medical/health misinformation during outbreaks and (2) reveal the needs and future directions for the development of new protocols that might contribute to the assessment and control of information quality in future infodemics.

## Methods

A systematic review was conducted to explore the determinants of infodemics during disease outbreaks. We focused on health misinformation on epidemics and pandemics that have been widely reported and commented in new and traditional media. This systematic review followed the Preferred Reporting Items for Systematic Reviews and Meta-Analyses (PRISMA) guidelines (28).

### Search Strategy for Study Identification

Databases were searched until December of 2019. According to the characteristic of the different search tools, specific research strategies were designed for Scopus, Medline, Embase, CINAHL, Sociological abstracts, Cochrane Library, plus gray literature. The searches were limited to the period from 01/01/2002 to 20/12/2019. The search terms were related with three basic dimensions: (1) epidemics; (2) misinformation; (3) internet and social media.

Specifically, we used the following search strategy and search terms: (“opinion” OR “opinions” OR “information” OR “misinformation” OR “rumor” OR “rumor” OR “rumors” OR “rumors” OR “gossip” OR “hoax” OR “hoaxes” OR “urban legend” OR “urban legends” OR “myth” OR “myths” OR “fallacy” OR “fallacies”) AND (“epidemics” OR “pandemics” OR “ebola” OR “ebola virus” OR “Ebola virus disease” OR “EVD” OR “zika” OR “zika virus” OR “zika fever” OR “H1N1” OR “Influenza A Virus” OR “Influenza in Birds” OR “avian flu” OR “avian influenza” OR “SARS” OR “severe acute respiratory syndrome”) AND (“online” OR “internet” OR “social media” OR “world wide web” OR “www” OR “social networks” OR “twitter” OR “facebook” OR “youtube” OR “whatsapp” OR “instagram” OR “forums”). The search of free words was complemented with MeSH terms on “information seeking behavior,” “epidemics,” “internet,” “social media” (additional information on the search strategy can be found in the Supplementary Table 1).

### Inclusion and Exclusion Criteria

According to the multidisciplinary nature of our research objective, in this systematic review we collected studies focused in the study of determinants of infodemics during disease outbreaks, drawing on both quantitative and qualitative methods. We focus on published research articles, written in English language, addressing the problem of health/medical misinformation during the periods of epidemics and pandemics. We included different methodological perspectives: (a) Quantitative research: studies based on experimental research and survey methods focused in the analysis of health misinformation through social media and the internet. In this section, we also included studies based on quantitative content analysis techniques; (b) Qualitative research: studies focused on preferences and/or individual predisposition to certain messages or sources of (mis)information through these new communicative channels, and qualitative studies based on the thematic analysis of misinformation; (c) Computational methods: studies based on more innovative approaches using computational methods for the study of complex processes of social contagion using text mining techniques, big data, machine learning algorithms, simulations, and social networks analysis.

We chose studies related to outbreaks that have been widely discussed through social media platforms and the Internet (e.g., SARS, H1N1, Ebola, Zika virus, etc.,). Studies focused on non-epidemic diseases, related to vaccination or based on information from other channels (i.e., traditional mass media) were excluded. According to the type of documents we excluded the following: abstracts, doctoral theses, editorials, press articles, commentaries and journal letters, and book reviews.

### Studies Quality Assessment

To assess the quality of the selected studies we used two instruments: one for quantitative studies and the other for qualitative ones (29, 30). The tool for assessment of quantitative studies included 10 items: (1) Did the study address a clearly focused issue?, (2) Did the authors use an appropriate method to answer their question?, (3) Was the study population clearly specified and defined?, (4) Were measures taken to accurately reduce measurement bias?, (5) Were the study data collected in a way that addressed the research issue?, (6) Did the study have enough participants to minimize the play of chance?, (7) Did the authors take sufficient steps to assure the quality of the study data?, (8) Was the data analysis sufficiently rigorous?, (9) How complete is the discussion?, and (10) To what extent are the findings generalizable to other international contexts? (where (0) “Can't tell”, (1) “poor”, (2) “fair”, (3) “good”).

The tool for assessment of qualitative studies included eight items: (1) Were steps taken to increase rigor in the sampling?, (2) Were steps taken to increase rigor in the data collected?, (3) Were steps taken to increase rigor in the analysis of the data? (where (0) “No, not at all/ Not stated/Can't tell,” (1) “Yes, a few steps were taken,” (2) “Yes, several steps were taken,” (3) “Yes, a fairly thorough attempt was made”), (4) Were the findings of the study grounded in/supported by the data? [(1) “Limited grounding/support,” (2) “Fair grounding/support,” (3) “Good grounding/support”], (5) Please rate the findings of the study in terms of their breadth and depth [(0) “Limited breadth or depth,” (1) “Good/fair breadth but very little depth,” (2) “Good/fair depth but very little breadth,” (3) “Good/fair breadth and depth”], (6) To what extent does the study privilege the perspectives and experiences of health care professionals and population health? [(0) “Not at all,” (1) “A little,” (2) “Somewhat,” (3) “A lot”], (7) Overall, what weight would you assign to this study in terms of the reliability/trustworthiness of its findings? [(1) “Low”, (2) “Medium,” (3) “High”], (8) What weight would you assign to this study in terms of the usefulness of its findings for this review? [(1) “Low,” (2) “Medium,” (3) “High”].

## Results

### Selection of the Studies

**Figure 1:**
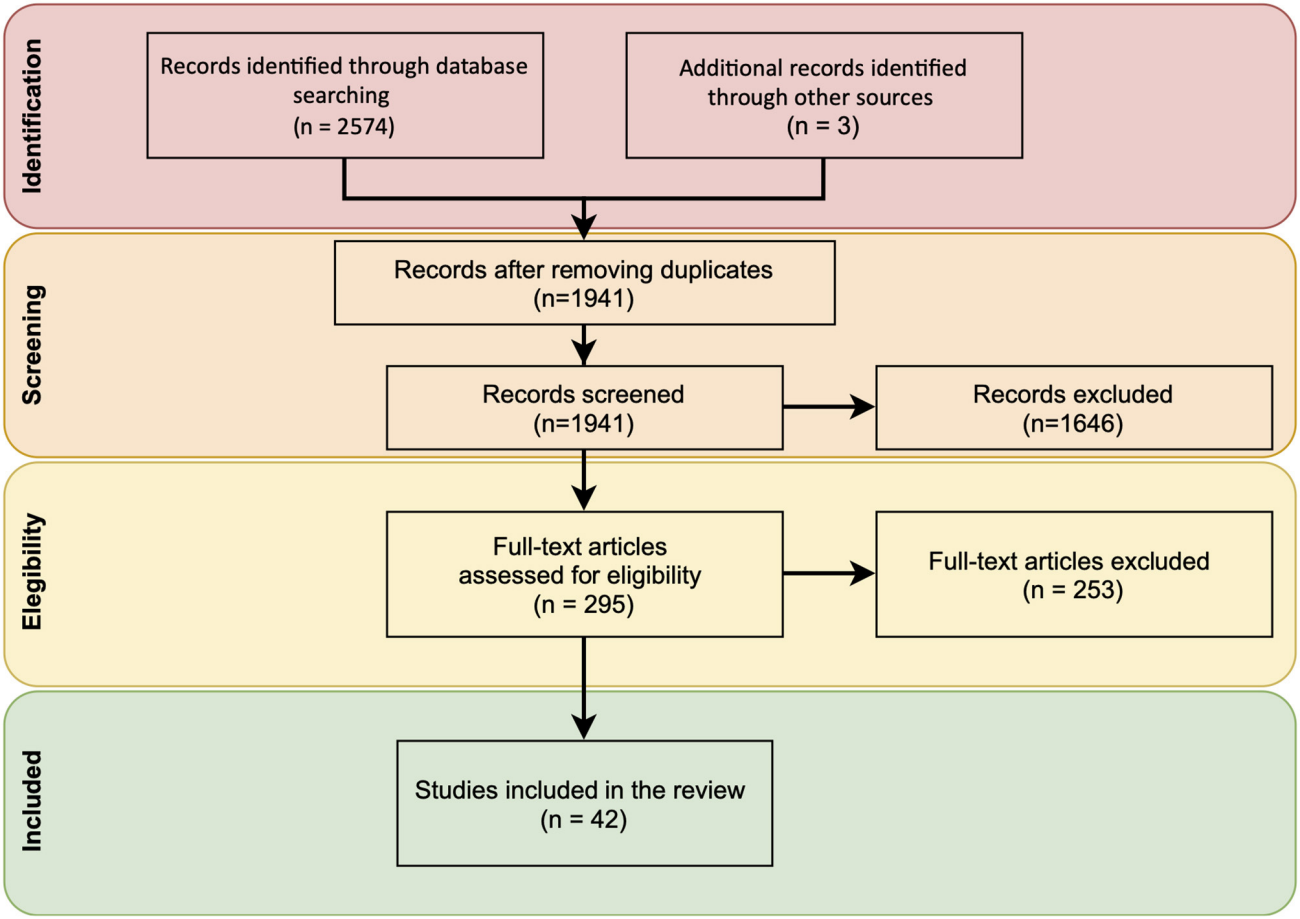
Flow diagram of studies through the review process.

After searching the main biomedical databases and consulting key papers, 2,577 references were retrieved. Then, duplicated records were removed and 1,941 references were screened. In the next stage, the authors independently carried out a full-text selection process for inclusion. Discrepancies were shared and resolved by mutual agreement. Finally, after reviewing their titles and abstracts, 295 references were assessed by full-text. The main reasons for excluding references were that studies did not address misinformation during epidemics or pandemics. Finally, 42 studies were included in the review for further assessment (Figure 1).

The reliability of the study selection process was assessed by the two of the authors using Cronbach's Alpha. The final selection showed high inter-rater reliability (α = 0.97).

### Characteristics of the Studies

Included papers were focused on nine communicable disease topics: Dengue (1), Ebola (15), Generic diseases (1), H1N1 (11), H7N9 (1), Poliomyelitis (3), SARS (1), and Zika (9). Most studies were selected from the period 2011–2019, the second decade of XXI century when the use of social media platforms widely spread. The main sources under study were social media (52.4%) and online surveys (19.0%). Most studies were based on quantitative methods (66.7%), but we also found qualitative (21.4%) and mixed methods studies (11.9%). Considering that selected studies were based on opinions and content generated in the Internet, a relevant part of the works have no particular geographic area of study (40.5%). The main characteristics of the studies identified are described in Table 1.

**Table 1:** Number of documents and its attributes.

**Attribute**	**Category**	**No. Studies**	**Percentage**
Disease	Dengue	1	2.4
	Ebola	15	35.7
	Generic	1	2.4
	H1N1	11	26.2
	H7N9	1	2.4
	Polio	3	7.1
	SARS	1	2.4
	Zika	9	21.4
Internet sources	Social media (Twitter, Facebook…)	22	52.4
	Forums/blogs/websites	3	7.1
	Mixed Sources	2	4.8
	Online news	3	7.1
	Online survey	8	19.0
	Other (reviews, qualitative data.)	4	9.5
Methods	Qualitative	9	21.4
	Quantitative	28	66.7
	Mixed methods	5	11.9
Data analysis	Content analysis	13	31.0
technique	Survey/scales	13	31.0
	Experimental/computational analysis	15	35.7
	Qualitative synthesis or review	1	2.4
Country	Australia	1	2.4
	Canada	4	9.5
	France	1	2.4
	Germany	1	2.4
	Italy	1	2.4
	Israel	2	4.8
	Netherlands	1	2.4
	Nigeria	1	2.4
	USA	9	21.4
	UK	4	9.5
	Worldwide	17	40.5
Total		42	100.0

According to the studies, different determinants of infodemics have been identified: (1) information sources (i.e., sender); (2) online communities' structure and consensus (i.e., receivers); (3) communication channels (i.e., mass media, social media, forums, etc.,); (4) messages content (i.e., quality of information, sensationalism, etc.,); and (5) the health emergency context (e.g., social consensus, health emergencies, public opinion, etc.,).

#### Health Misinformation Sources

Health misinformation can propagate through influencers or well-positioned individuals that may act as distractors or judges in specific social networks (31). In addition, certain individual profiles such as those belonging to anti-vaccine groups look for alternative information and treatments online (32), and this argument can also be applied to low educated groups, people having a low digital health literacy (33), or just poor general knowledge on specific diseases such as Ebola or Zika (34). However, gaps and biases in current scientific knowledge have also been found (35); therefore misinformation can be also derived from poor quality scientific knowledge (e.g., misleading scientific papers and/or studies based on preliminary or biased results) which can be subsequently spread through mass and social media. In fact, recent studies have demonstrated a high correlation between social media tweets/posts and news articles information (36). Therefore, traditional media can also contribute to the wrong interpretation of existing scientific evidence and thus to the massive spreading of poor-quality messages that often echoed in social media (24).

#### Online Communities' Structure

Among the assessed studies we have found that opinion polarization and echo chamber effects can increase (mis)information divides due to the homophily between social media users, but also the resistance to evidence-based knowledge and behavioral change between community members (37, 38). For instance, in the context of social media such as Facebook or Twitter, people tend to spread either good or bad information to their friends. Although expert knowledge from health authorities is also widely distributed in these platforms, misleading health contents propagate and reverberate among relatively closed online communities which ultimately reject expert recommendations and research evidence. Again, this is the case of anti-vax groups that increase vaccine hesitancy through the promotion of public debates around the medical benefits, ethical, and legal issues related to vaccination. Consequently, health misinformation and infodemics can spread easily among certain online communities composed by individuals with similar beliefs and interests (39), and this includes the scientific community which is constantly exposed to trending research topics (35).

#### Communication Channels

According to the selected studies, although mass media can also contribute to the propagation of poor-quality information during public health emergencies, social media seem to be an ideal channel to spread anecdotal evidence, rumors, fake news, and general misinformation on treatments and existing evidence-based knowledge about health topics (40, 41). For example, it is easy to find poor quality and misleading information on the MMR vaccine (and its relationship with autism) both in the internet and social media such as Twitter, Facebook, or YouTube (31). In general, studies demonstrated that the number of high-quality websites was profoundly limited (42), although today we can also find evidence that certain online sources may also enhance health literacy (43) and self-efficacy in fulfilling treatments of specific health information seekers (e.g., chronic patients looking for health solutions) (8).

#### Messages Content

Alarmist, misleading, shorter messages, and anecdotal evidence seem to have a stronger impact on the spread of health-related misinformation on epidemiological topics, which can develop and reproduce infodemics. For instance, misleading posts about the Zika virus have been found to be more popular than the posts disseminating accurate information (25, 27, 44). The narratives of misinformation and misleading contents commonly induce fear, anxiety, and mistrust around government and health institutions (45). However, shorter messages have also been found effective for promoting peoples' health, specifically in vaccination campaigns aimed to fight vaccine hesitancy and thus promoting vaccination behavior (46). Twitter, as a microblogging service, can be really fast to propagate evidence-based knowledge on health, and YouTube videos can be easily used to increase knowledge on specific epidemiological topics (or related concerns) that can be difficult to transmit through other communication channels (47).

#### Health Emergency Context

Regarding the emergency context of situations related to epidemiological concerns, and particularly during pandemics, studies showed ambivalent findings. Although, under health crises alarmist or misleading messages in social media might derive in risky behaviors (e.g., use of alternative medicines, miracle remedies, dangerous treatments, vaccination rejection, etc.,), these social platforms have also been found helpful to manage health information uncertainty and health behaviors through the rapid propagation of evidence-based knowledge which is needed to control specific diseases (46). During emergency circumstances, platforms such as Twitter, Facebook, Instagram, or YouTube have the potential for enhancing educational content on the etiology and prevention of disease, but also for spreading health misinformation (48–52). Therefore, during health crisis contexts, social media can be used to both promote and combat health misinformation.

On the other side, traditional media can also contribute to the propagation and reproduction of misleading contents, which may ultimately affect the development and prolongation of infodemics (53, 54). In this vein, recent studies point to the need to avoid alarmism and sensationalism of messages in order to avoid public misconceptions (55) and the reproduction of these messages through social media (24). These results are summarized in Table 2.

**Table 2:** Description of the studies assessed.

**Author(s)**	**Year**	**Study design**	**Disease**	**Main outcomes**	**Misinformation type**	**Internet source**	**Meth. quality**
Atlani-Duault et al.	2015	Qualitative design	H1N1 virus	Identify online rumors on the H1N1 which was absent from the discussions in mainstream mass media (e.g., hidden motives of governments, and pharma companies…).	Online rumors on H1N1	Blogs and websites Mass media and comments	Poor
Chimuanya and Ajiboye	2016	Qualitative design	Ebola virus	Humorous posts may be useful in tackling social problems, however, online readers should be cautious with the interpretation of content of these messages.	Humor memes (visual jokes.)	Facebook	Poor
Covolo et al.	2013	Mixed design	H1N1 virus	The majority of the websites analyzed had a positive/neutral attitude toward flu vaccination and overall, they provided satisfactory information.	Classification of information (pro-neutral-adverse) according WHO reference.	Websites	Good
Charles-Smith et al.	2015	Qualitative design	All disease outbreak	Studies on the use of social media to support public health practice has identified many gaps and biases in current knowledge.	Misinformation on different health related topics.	Social media	Good
Mollema et al.	2015	Mixed design	Measles	High correlation between tweets and news articles information. The monitoring of online (social) media might be useful for improving communication policies and increase vaccination acceptability.	Messages on measles in Twitter and Online news articles.	Measles-related tweets (Twitter and other sources) Online news articles	Good
Househ	2016	Cross-sectional	Ebola virus	Relationship between electronic news media publishing and Twitter activity around significant events such as Ebola. This information could be used to design social media campaigns. Electronic news media reports have influenced the number of social media discussions.	Online information on Ebola virus.	Twitter News media	Fair
Sharma et al.	2017	Qualitative design	Zika	Misleading posts are far more popular than the posts dispersing accurate, relevant public health information about the disease.	Misinformation on Zika virus	Facebook	Good
Henrich and Holmes	2011	Mixed design	H1N1 virus	News' commenters may have played a significant role in their decision-making about whether or not to receive the H1N1 vaccine.	Misinformation on MMR vaccine	News articles and comments	Good
Rao et al.	2012	Cross-sectional (survey)	Dengue	The number of high-quality websites was limited, but those sites had high information credibility and were more relevant. Need to educate consumers on how to find and recognize valid health information on the Internet will promote better decision making.	Misinformation on Dengue	Survey on the quality of internet websites and forums	Fair
Rubsamen et al.	2015	Cross-sectional (survey)	Ebola virus	Population demonstrated poor knowledge about the transmission of Ebola and about the actual risks.	Misinformation on Ebola virus	Survey on the knowledge of Ebola and its risks	Good
Seeman et al.	2010	Cross-sectional (survey)	H1N1 virus	Need to use real-time web analytic tools to detect misinformation on H1N1 vaccines and other health-related issues.	Anti-vaccine sentiment	Websites and blog posts with anti-vaccine sentiment	Good
Ballester et al.	2011	Cross-sectional (survey)	Poliomyelitis	The study evidence the poor quality of websites related with polio. This is relevant taking into account that internet users do not generally evaluate the quality of information online.	Poliomyelitis information on websites	Survey on websites content	Good
Vos and Buckner	2016	Mixed design	H7N9 virus	The study show that a high proportion of messages contained sense making information. However, few tweets contained efficacy information that would help individuals respond to the crisis appropriately.	Messages containing information on H7N9 virus	Twitter	Good
Hill et al.	2011	Observational study	H1N1 virus	The prevalence of non-authoritative information on supplements and the increasing number of searches for these pages suggest that the public is interested in alternatives to traditional prevention and treatment of H1N1. The quality of this information is often questionable.	Websites containing information on natural supplements for H1N1 virus	Websites, blogs…	Good
Nagpal et al.	2015	Observational study	Ebola virus	YouTube videos presenting clinical symptoms of infectious diseases during epidemics are more likely to be included in the high relevance group and influence viewers behavior.	Videos on Ebola virus	Youtube	Good
Towers et al.	2015	Experimental design	Ebola virus	High correlation between Ebola-related news video, tweets and Internet searches. Between 65 and 76% of the variance in all samples is described by the news media. Mass media sets the thematic agenda.	Traditional mass media and its correlation with Twitter discussion	Twitter	Good
Koralek et al.	2016	Observational study	Ebola virus	Information sources are likely to influence students' knowledge, attitudes, beliefs, and stigma relating to EVD. This study contains crucial insight for those tasked with risk communication to college students. Need to develop effective strategies to achieve a comprehensive knowledge of EVD and future public health threats.	Opinions and beliefs of students about Ebola virus	None	Good
Bessi et al.	2016	Quantitative design	None (Ebola is mentioned)	Facebook users tend to be very polarized between those that access to good and poor information. People prefer to follow ideas coming from people with their same ideas (homophily). People who believe in rumors will likely contribute to the spread of misinformation.	General health misinformation	Facebook	Good
Gesser-Edelsburg et al.	2017	Qualitative design	Poliomyelitis	We need additional evidence on the effect of the impact of medical information online. Exposure to a wider variety of sources may enhance health literacy, resulting in a better understanding of information needed to make informed decisions.	Misinformation on Polio/Opinions on Polio	Websites, blogs, forums Facebook posts	Good
Lazard et al.	2016	Quantitative design	Ebola virus	Social media text mining provides a valuable tool that can be used quickly and efficiently to improve public health communication efforts by collecting and identifying prevalent themes of public concern.	Opinions and concerns related to Ebola	Twitter	Good
Chesser et al.	2016	Quantitative design	Ebola virus	Results from this study highlight the need to improve health communication training and further evaluate the quality of health information dissemination *via* all communication sources.	Opinions about media coverage on Ebola virus	None	Fair
Ashbaugh et al.	2013	Quantitative design	H1N1	This study suggest that public health officials should not only discuss the dangers of the pandemic but also (i) take additional steps to reassure the public about the safety of vaccines and (ii) monitor the information disseminated over the Internet rather than relying on the more traditional mass media.	Web-based survey	None	Good
Crook et al.	2016	Quantitative design	Ebola virus	The major themes identified: etiology of Ebola, policy, the environment, spread and scope of the disease, fear and anxiety from the public, and misinformation. Practical implications of these findings include encouraging government and emergency health response organizations to prepare educational messages and materials in advance that detail responses to common questions, such as transmission and symptoms.	Misinformation on Ebola virus	Twitter	Fair
Nerlich and Koteyko	2012	Qualitative design	H1N1	This study evidence the critical need for informed journalism (WHO guidelines) to avoid alarmism and sensationalism and misinformation of the public. Existence of parallel online discourse that complement official information and the need to control this information.	Mass media communication on H1N1	Mass media articles	Good
Godinho et al.	2016	Quantitative design	H1N1	This study has demonstrated that shorter messages are more effective in promoting peoples' intentions to be vaccinated. Its results suggest that messages should communicate information on the new strain of virus and that virtually anyone is at-risk, and on vaccine effectiveness and safety tests.	Opinions and information on H1N1	None	Good
Chew and Eysenbach	2010	Quantitative design	H1N1	This study illustrates the potential of using social media to conduct “infodemiology” studies for public health. 2009 H1N1-related tweets were primarily used to disseminate information from credible sources, but were also a source of opinions and experiences. Tweets can be used for real-time content analysis and knowledge translation research, allowing health authorities to respond to public concerns.	Opinions and information on H1N1	Twitter	Good
Jardine et al.	2015	Quantitative design	SARS, H1N1	People are increasingly using multiple sources of health risk information, presumably in a complementary manner. Subsequently, although using online media is important, this should be used to augment rather than replace more traditional information channels. Efforts should be made to improve knowledge transfer to health care professionals and doctors and provide them with opportunities to be more accessible as information sources.	Opinions on SARS and H1N1 pandemics	None	Good
Basch et al.	2015	Quantitative design	Ebola virus	With 1 billion unique users a month, YouTube has potential for both enhancing education and spreading misinformation.	Youtube videos on Ebola virus	Youtube	Good
Orr et al.	2016	Qualitative design	Poliomyelitis	Health officials and experts need to be accessible on social media, and be equipped to readily provide the information, support and advice the public is looking for in order to avoid the spread of poor-quality information.	Comments on social media	Social media platforms (and Facebook) Online mass media articles	Good
Wasim et al.	2019	Qualitative design	H1N1	Informal terms used to refer to disease outbreaks such as swine flu should be avoided because they lead to confusion. Twitter data could be utilized by library professionals for developing a better understanding of public views on health-related topics.	Opinions and information on H1N1	Twitter	Good
Bora et al.	2018	Quantitative design	Zika	Misleading videos were more popular and could potentially spread misinformation. Curation/authentication of health information in online video platforms is necessary.	Youtube videos on Zika virus	Youtube videos	Good
Bragazzi et al.	2017	Quantitative design	Zika	The majority of queries concerned the symptoms of the Zika virus, its vector of transmission, and its possible effect to babies, including microcephaly. No statistically significant correlation was found between novel data streams and global real-world epidemiological data. At country level, a correlation between the digital interest toward the Zika virus and Zika incidence rate or microcephaly cases has been detected.	Opinions and information on Zika virus	Tweets, Google Trends, Google News, YouTube, and Wikipedia search queries.	Good
Daughton and Paul	2019	Quantitative design	Zika	Differences in the demographics, social networks, and linguistic patterns of 1,567 individuals identified as changing or considering changing travel behavior in response to Zika as compared with a control sample of Twitter users. Significant differences between geographic areas were found in the United States, significantly more discussion by women than men, and some evidence of differences in levels of exposure to Zika-related information.	Opinions and information on Zika virus	Tweets about Zika virus	Good
Liang et al.	2019	Quantitative design	Ebola	Broadcasting was the dominant mechanism of information diffusion of a major health event on Twitter. Although, both influential users and hidden influential users can trigger many retweets, recognizing and using the hidden influential users as the source of information could potentially be a cost-effective communication strategy for public health promotion.	Spreading information and emotions in Twitter about Ebola	Tweets about Ebola	Good
Mamidi et al.	2019	Quantitative design	Zika	10 topics for each sentiment category were identified using topic modeling, with a focus on the negative sentiment category.	Identifying key topics on Twitter	Tweets about Zika	Good
Miller et al.	2017	Quantitative design	Zika	Five topics for each category were found and discussed, with a focus on the symptom's category. The two-stage classifier was able to identify relevant tweets to enable more specific analysis, including the specific aspects of Zika that were being discussed as well as misinformation being expressed.	Identifying key topics on Twitter	Tweets about Zika	Good
Morin et al.	2018	Mixed design	Ebola	The results confirm the significant role played by mainstream media in disseminating information, media did not create the debate around the sexual transmission of Ebola and Twitter does not fully reflect mainstream media contents.	Information and emotions in Twitter about sexual transmission of Ebola	Tweets about Ebola	Good
Roberts	2017	Quantitative design	Ebola	Corresponding public sentiments about Ebola were reflected in the policy responses of the international community, including violations of the International Health Regulations and the treatment of potentially exposed individuals. The digitally networked global public may have influenced the discourse, sentiment, and response to the Ebola epidemic.	Information and emotions in different social media platforms	Stories from different social media platforms about Ebola	Good
Seltzer et al.	2017	Qualitative design	Zika	Instagram can be used to characterize public sentiment and highlight areas of focus for public health, such as correcting misleading or incomplete information or expanding messages to reach diverse audiences.	Opinions toward Zika virus	Images and stories about Zika virus on Instagram	Good
Stefanidis et al.	2017	Quantitative design	Zika	The spatiotemporal analysis of Twitter contributions reflects the spread of interest in Zika from its original hotspot in South America to North America and then across the globe. Tweets about pregnancy and abortion increased as more information about this emerging infectious disease was presented to the public and public figures became involved in this.	Opinions and information on Zika virus	Tweets about Zika virus	Good
Van Lent	2017	Quantitative design	Ebola	Analyses based on 4,500 tweets revealed that increases in public attention to Ebola co-occurred with severe world events related to the epidemic, but not all severe events evoked fear. As hypothesized, Web-based public attention and expressions of fear responded mainly to the psychological distance of the epidemic.	Epidemiological data and its correlation with Twitter data	Epidemiological data and media data (tweet volume and key events reported in the media)	Good
Vijaykumar et al.	2018	Qualitative design	Zika	The most talked about theme was Zika transmission (~58%). News media, public health institutions, and grassroots users were the most visible and frequent sources and disseminators of Zika-related Twitter content. Grassroots users were the primary sources and disseminators of conspiracy theories.	Opinions and information about Zika virus	Zika-related tweets	Good

### Quality Assessment of Included Studies

Most of the studies analyzed met the basic criteria of methodological quality. Of the total number of studies evaluated in this review, 85.7% (36) had a good quality assessment, 9.5% (4) had a moderate quality assessment and 4.8% (2) received a poor assessment (mainly due to the impossibility to complete certain methodological criteria). Therefore, the risk of bias is relatively low. Scores for the items of the used quality assessment instrument are included in the Supplementary Table 2.

## Discussion

Although the concept of infodemics has recently been conceptualized to explain the difficulties for screening the current overabundance of information on the SARS-CoV-2 and the COVID-19 disease, our review demonstrates that this problem is not new since the same dynamics have occurred in previous pandemics (24–27, 32, 37, 44–47, 49–51, 56–77). These studies show that infodemics are closely related to the rapid and free flow of (mis)information through the Internet, social media platforms and online news (78). Although, these new media present a high potential to massively spread evidence-based knowledge, the speed and lack of control of health information contents (even coming from the scientific community) can easily undermine basic standards for trustworthy evidence and increase the risk of bias in research conclusions (79).

As many studies hypothesize, misinformation can easily propagate through social media platform such as Twitter, Facebook, YouTube, or WhatsApp. Misconceptions about H1N1, Zika, or Ebola pandemics are common on social media, but this unofficial narrative is generally absent from the discussions in mainstream mass media (56). This trend can also be observed on rumors regarding the association between vaccination and autism (particularly referred to the MMR vaccine), which generally proliferate on social media (41, 80) or, for instance, among messages around conspiracy theories of governments and pharmaceutical companies that are usual in the virtual environment of social media communities (81). However, studies also demonstrate that social media may be very useful for fighting misinformation during public health crisis (42, 74). Therefore, these social platforms have also an immense capacity to propagate evidence-based health information which might help for mitigating rumors and anecdotal evidence (41). In fact, social media such as Twitter or Facebook have been found to be particularly effective tools in combating rumors, as they can complement and support general information from the mainstream media (53).

These findings demonstrate that these tools have also a huge potential to fight health misinformation through the promotion of health-related educational content and material oriented to solve common questions such as those related to transmission, symptoms, and prevention, as long as they are properly coordinated by specific programmes and interventions from governments and health organizations (66). Nevertheless, taking into consideration that anecdotal evidence and rumors seem to be more popular in social media than evidence-based knowledge (44), public health authorities should find alternatives ways to reach health-information seekers while combating–both the official and unofficial–sources of misinformation, but in particular unauthorized and suspicious social media accounts whose affiliation is not clearly defined (57, 82). In this context, it is needed to consider the particular case of “expert patients” that might ambivalently promote accurate health information and anecdotal experiences (83). Although patients' expertise might be a valuable resource for understanding the patient's perspective, the health professionals are better prepared to confront uncertainty situations and identify concrete solution that would lead to positive health outcomes (84). In any case, it is fundamental to understand that during periods of health emergencies such as the COVID-19 pandemic, even researchers and health professionals can contribute to obscuring the scientific evidence since the hasty search for solutions can lead to biased conclusions (85). This argument also applies to opinion leaders. For example, after Trump suggested in the media that disinfectants could be used to treat Coronavirus, the Georgia Poison Center revealed that two men guzzled cleaning solution over the next weekend in misguided attempts to prevent catching COVID-19.

In order to understand how misinformation might propagate among social media, it is relevant to introduce the distinction between “simple” and “complex contagion” in the framework of opinion spreading in social networks. The fundamental difference between this concept is that while simple contagion depends on network connectivity (e.g., epidemiologic contagion of disease), the process of complex contagion of opinion and ideas requires multiple reinforcement that are based on legitimacy in online communities and normative social consensus (86, 87). Consequently, the effective propagation of misinformed ideas and anecdotal evidence related with health topics depends on the connectivity between social media users, but in particular on the social legitimacy to share these ideas in different normative contexts (88). Therefore, the processes of health misinformation spreading that might contribute to the development of infodemics through social media are also related to the social consensus between groups and the social structures among their online communities (i.e., the degree of connectivity between users and the topological configuration of their social network) (89).

The diversity of studies that we have found indicates the general complexity of delimiting evidence-based knowledge during health emergency contexts in which, due to the very situation of uncertainty and either because of an overabundance of evidence or because of an increase in false news, disinformation tends to emerge naturally (85, 90). Although the negative impact of health misinformation on public health is widely assumed in different studies (5), there is still little evidence on three central questions: (a) how to measure the prevalence of health misinformation in social media; (b) how to analyse the influence between exposure to health-related misinformation and health behaviors and outcomes; and in particular (c) how to reduce the spreading and exposure to health misinformation through specific interventions and programmes (5). Thus, understanding the phenomenon of complex contagion of misinformation during health emergencies, it will be essential to explain the emergence and reproduction of future infodemics.

Studied selected in this systematic review described different measures to combat health misinformation in mass and social media: (1) to develop formative programs for professional intervention and better knowledge transfer (91); (2) to improve health-related contents in mass media by using existing (peer-reviewed) scientific evidence (78); (3) to promote the development of multimedia products (videos, images, tutorials, infographics, etc.,) from trustworthy sources like academic institutions and health organizations (i.e., research evidence should be particularly oriented to the public so that scientific knowledge can produce social impact) (27); (4) to develop coordinated information campaigns between the media and health authorities, specifically during periods of health crisis (53); (5) to increase (digital) health literacy of health-information seekers (42, 48, 66); and (6) to develop new tools and information systems for health misinformation monitoring and health evidence quality assessment (61, 92). Considering that the highly changing and dynamic ecosystem were infodemics rise and evolve, the action of knowledge developers and spreaders is central for the control of fake news, anecdotal evidence, false, and misleading contents. Therefore, health organizations, reputed hospitals, universities, research institutes, mass media, and social media companies may play a more proactive role in the fight of health mis/disinformation. In general, scientific knowledge should have larger presence and visibility in the “information societies,” but particularly during contexts of health emergencies–as the COVID-19 pandemic–were overabundance of information might induce contradictory views, population fear, and undesired social consequences such as rejection of new governmental rules, lack of trust in health authorities, denial of expert advices, and vaccine and treatment hesitancy, among others social side effects.

### Limitations and Future Directions

We found some limitations when conducting our systematic review. First, due to the novelty of the research topics, we detected a great heterogeneity of results in the articles included. Most studies selected were observational study designs based in survey methods or descriptive content analysis applied to different population groups (students, general population, or platform users), but we also found other works based on computational methods (i.e., including online data or social media data) and even on qualitative evidence. Second, considering the idiosyncrasies of every platform (i.e., microblogging services, video sharing, etc.,) we will need additional studies to obtain platform-oriented conclusions and further methodological insights on how to effectively combat the determinants health misinformation during health disease outbreaks and subsequent infodemics across different social media platforms.

Finally, it is worth to mention that as authors are editing this paper, the COVID-19 infodemic is currently taking place. Therefore, the consequences of the misinformation regarding symptoms of the new disease, the possible treatments and public health strategies to cope with the new pandemic are still unknown. Our text search covered up to December 2019 and did not capture studies on the COVID-19. Nevertheless, it emphasizes the existence of a problematic and recurrent social phenomenon that reinforce the need to collate and analyse evidence about the relationship between disease outbreaks and health misinformation. Thus, the COVID-19 pandemic confirms the relevance to internationally address this research topic from a multidisciplinary approach that could integrate existing evidence of different but complementary research fields.

## Conclusion

Our systematic review provides a comprehensive characterization of the determinants of infodemics and describes different measures to combat health misinformation during disease outbreaks. The clarity of the health promotion messages has been proven essential to prevent the spread of a particular disease and to avoid potential risks, but it is also fundamental to understand the network structure of social media platforms and the context where misinformation and/or misleading evidence might dynamically evolve. Therefore, in order to prevent future infodemics, special attention will need to be paid both to increase the visibility of evidence-based knowledge generated by health organizations and academia, and to detect the possible sources of mis/disinformation. In any case, this fight against health misinformation will not be possible without the help of mass media and social media companies, particularly the latter since these platforms have a high penetration in our societies and, consequently, the capacity to work on the health literacy of the population. We must consider that the battle against misinformation is not exclusive to the health field. Misinformation is also rooted in the political and economic system of our societies, but –as we can observe in these days of pandemic– it is probably during the processes of social and health emergencies when the damage to our way of life may be greater.

## Author Contributions

JA-G contributed conception of the study. AR-G and VS-L contributed in the selection of studies, the screening process, and grading of evidence. JA-G and AR-G wrote the first draft of the manuscript. All author contributed in the interpretation of results, have contributed substantially to the development of the paper, and approved the final version of the manuscript for its submission to this journal.

## Conflict of Interest

The authors declare that the research was conducted in the absence of any commercial or financial relationships that could be construed as a potential conflict of interest.
