# Supplementary material for: Determinants of Infodemics During Disease Outbreaks: A Systematic Review
Source: Front Public Health. 2021 Mar 29;9:603603. doi: 10.3389/fpubh.2021.603603 (PMC8039137; doi:10.3389/fpubh.2021.603603)
Supplement: Supplementary file 1 [file Table_1.DOCX]

**Supplementary Table 1**. Search terms and results

| **Blocks** |  | **Searches – Medline (Ovid)** | **Results** |
| --- | --- | --- | --- |
| Misinformation | 1 | MeSH: exp Information Seeking Behavior/ OR exp propaganda/ OR exp Deception/ OR exp Bias (Epidemiology) / exp Health Knowledge, Attitudes, Practice / | 172692 |
|  | 2 | Free terms: ("opinion" OR "opinions" OR "information" OR "misinformation" OR "rumour" OR "rumor" OR "rumours" OR "rumors" OR "gossip" OR "hoax" OR "hoaxes" OR "urban legend" OR "urban legends" OR "myth" OR "myths" OR "fallacy" OR "fallacies").ti,ab | 1139710 |
|  | 3 | 1 OR 2 | 1281070 |
| Crisis / outbreak / Diseases | 4 | MeSH: exp Hemorrhagic Fever, Ebola/ OR exp Ebolavirus/ OR exp Zika virus/ OR exp Zika Virus Infection OR exp Influenza A virus/ OR exp Influenza in Birds/ OR exp SARS Virus/ OR exp Disease Outbreaks | 144823 |
|  | 5 | Free terms: ("epidemics" OR "pandemics" OR "ebola" OR "ebolavirus" OR "Ebola virus disease" OR "EVD" OR "zika" OR "zika virus" OR "zika fever" OR "N1H1" OR "Influenza A Virus" OR "Influenza in Birds" OR "avian flu" OR "avian influenza" OR "SARS" OR "severe acute respiratory syndrome").ti,ab | 37248 |
|  | 6 | 3 OR 4 | 144823 |
| Internet / Social media | 7 | MeSH: exp internet/ or exp social networking | 74063 |
|  | 8 | Free terms: ("online" OR "internet" OR "socia media" OR "world wide web" OR "www" OR "social networks" OR "twitter" OR "facebook" OR "youtube" OR "whatsapp" OR "instagram" OR "forums").ti,ab | 116553 |
|  | 9 | 5 OR 6 | 157833 |
|  | 10 | 3 AND 6 AND 9 | 498 |
|  | 11 | *Remove duplicates from* 10 | 422 |
