# Supplementary material for: Determinants of Infodemics During Disease Outbreaks: A Systematic Review
Source: Front Public Health. 2021 Mar 29;9:603603. doi: 10.3389/fpubh.2021.603603 (PMC8039137; doi:10.3389/fpubh.2021.603603)
Supplement: Supplementary file 2 [file Table_2.DOCX]

**Supplementary Table 2**. Scores of the items measured through the methodological quality assessment.

| ***Author(s)*** | ***Year*** | ***Q1*** | ***Q2*** | ***Q3*** | ***Q4*** | ***Q5*** | ***Q6*** | ***Q7*** | ***Q8*** | ***Q9*** | ***Q10*** | ***MEAN*** | ***Columna1*** |
| --- | --- | --- | --- | --- | --- | --- | --- | --- | --- | --- | --- | --- | --- |
| L. Atlani-Duault, et al. | 2015 | 1 | 2 | 1 | 1 | 1 | 0 | 1 | 1 | NA | NA | 1.0 | Poor |
| L. Chimuanya and E. Ajiboye | 2016 | 1 | 1 | 1 | 1 | 1 | 0 | 1 | 1 | NA | NA | 0.9 | Poor |
| L. Covolo, et al. | 2013 | 3 | 3 | 2 | 3 | 3 | 3 | 3 | 3 | 3 | 3 | 2.9 | Good |
| L. E. Charles-Smith, et al. | 2015 | 3 | 3 | 3 | 3 | 3 | 3 | 3 | 3 | 3 | 3 | 3.0 | Good |
| L. Mollemaet al. | 2015 | 3 | 3 | 3 | 3 | 3 | 3 | 3 | 3 | 3 | 3 | 3.0 | Good |
| M. Househ | 2016 | 3 | 2 | 2 | 2 | 2 | 2 | 2 | 2 | 2 | 2 | 2.1 | Fair |
| M. Sharma, et al. | 2017 | 3 | 2 | 2 | 2 | 3 | 3 | 3 | 3 | 3 | 3 | 2.7 | Good |
| N. Henrich and B. Holmes | 2011 | 3 | 3 | 3 | 2 | 3 | 3 | 3 | 3 | 3 | 3 | 2.9 | Good |
| N. R. Rao, et al. | 2012 | 2 | 2 | 2 | 1 | 1 | 1 | 1 | 1 | 2 | 1 | 1.4 | Fair |
| N. Rubsamen, et al. | 2015 | 3 | 2 | 3 | 3 | 2 | 3 | 2 | 3 | 3 | 3 | 2.7 | Good |
| N. Seeman, et al. | 2010 | 3 | 3 | 3 | 2 | 2 | 3 | 2 | 3 | 3 | 3 | 2.7 | Good |
| R. Ballester, et al. | 2011 | 2 | 2 | 2 | 1 | 2 | 2 | 2 | 3 | 3 | 2 | 2.1 | Good |
| S. C. Vos and M. M. Buckner | 2016 | 3 | 3 | 3 | 2 | 3 | 3 | 2 | 2 | 3 | 2 | 2.6 | Good |
| S. Hill, et al. | 2011 | 3 | 3 | 3 | 3 | 3 | 3 | 3 | 3 | 3 | 3 | 3.0 | Good |
| S. J. S. Nagpal, et al. | 2015 | 3 | 2 | 3 | 2 | 2 | 3 | 3 | 3 | 3 | 3 | 2.7 | Good |
| S. Towers, et al. | 2015 | 3 | 3 | 3 | 3 | 3 | 3 | 3 | 3 | 3 | 3 | 3.0 | Good |
| T. Koralek, et al. | 2016 | 3 | 2 | 2 | 3 | 2 | 3 | 3 | 3 | 3 | 3 | 2.7 | Good |
| A. Bessi, et al. | 2016 | 3 | 3 | 3 | 2 | 3 | 3 | 3 | 3 | 3 | 3 | 2.9 | Good |
| A. Gesser-Edelsburg, et al. | 2017 | 3 | 2 | 3 | 2 | 2 | 1 | 2 | 3 | 3 | 2 | 2.3 | Good |
| A. J. Lazard, et al. | 2015 | 3 | 2 | 3 | 2 | 3 | 2 | 2 | 2 | 3 | 2 | 2.4 | Good |
| A. K. Chesser, et al. | 2016 | 3 | 3 | 3 | 1 | 1 | 1 | 2 | 2 | 3 | 1 | 2.0 | Fair |
| A. R. Ashbaugh, et al. | 2013 | 3 | 3 | 3 | 3 | 3 | 3 | 3 | 3 | 3 | 3 | 3.0 | Good |
| B. Crook, et al. | 2016 | 2 | 1 | 1 | 1 | 2 | 2 | 1 | 1 | 2 | 1 | 1.4 | Fair |
| B. Nerlich and N. Koteyko | 2012 | 3 | 3 | 3 | 3 | 3 | 2 | 3 | 2 | NA | NA | 2.8 | Good |
| C. A. Godinho, et al. | 2016 | 3 | 3 | 3 | 3 | 3 | 3 | 3 | 3 | 3 | 3 | 3.0 | Good |
| C. Chew and G. Eysenbach | 2010 | 3 | 3 | 3 | 3 | 3 | 3 | 3 | 3 | 3 | 3 | 3.0 | Good |
| C. G. Jardine, et al. | 2015 | 3 | 3 | 3 | 2 | 3 | 3 | 3 | 3 | 3 | 2 | 2.8 | Good |
| C. H. Basch, et al. | 2015 | 3 | 3 | 2 | 2 | 2 | 3 | 3 | 3 | 3 | 3 | 2.7 | Good |
| D. Orr, et al. | 2016 | 2 | 2 | 2 | 2 | 3 | 2 | 3 | 2 | NA | NA | 2.3 | Good |
| A. Wasim et al. | 2019 | 3 | 3 | 3 | 2 | 3 | 3 | 3 | 3 | 3 | 3 | 2.9 | Good |
| K. Bora et al. | 2018 | 3 | 3 | 3 | 2 | 3 | 3 | 3 | 3 | 3 | 2 | 2.8 | Good |
| N. L. Bragazzi et al. | 2017 | 3 | 2 | 3 | 2 | 2 | 1 | 2 | 3 | 3 | 2 | 2.3 | Good |
| A. Daughton and M. Paul | 2019 | 3 | 2 | 3 | 2 | 2 | 1 | 2 | 3 | 3 | 2 | 2.3 | Good |
| H. Liang et al. | 2019 | 3 | 3 | 2 | 2 | 2 | 1 | 2 | 3 | 3 | 2 | 2.3 | Good |
| R. Mamidi et al. | 2019 | 3 | 2 | 3 | 2 | 2 | 1 | 2 | 3 | 3 | 2 | 2.3 | Good |
| M. Miller et al. | 2017 | 3 | 2 | 3 | 2 | 2 | 1 | 2 | 3 | 3 | 2 | 2.3 | Good |
| C. Morin et al. | 2018 | 3 | 3 | 3 | 2 | 3 | 3 | 3 | 3 | 3 | 3 | 2.9 | Good |
| H. Roberts | 2017 | 3 | 3 | 2 | 3 | 3 | 3 | 3 | 3 | 3 | 3 | 2.9 | Good |
| E. Seltzer et al. | 2017 | 3 | 3 | 3 | 3 | 3 | 2 | 3 | 2 | NA | NA | 2.8 | Good |
| A. Stefanidis et al. | 2017 | 3 | 2 | 3 | 2 | 3 | 2 | 2 | 2 | 3 | 2 | 2.4 | Good |
| L. Van Lent | 2017 | 3 | 3 | 3 | 3 | 2 | 3 | 2 | 2 | 3 | 2 | 2.6 | Good |
| S. Vijaykumar et al. | 2018 | 2 | 2 | 2 | 2 | 3 | 2 | 3 | 2 | NA | NA | 2.3 | Good |
